# Mortality attributable to COVID-19 in nursing home residents: a retrospective study

**DOI:** 10.1007/s40520-021-01855-6

**Published:** 2021-04-24

**Authors:** Nicola Veronese, Ai Koyanagi, Vanni Stangherlin, Paola Mantoan, Marco Chiavalin, Florina Tudor, Gianfranco Pozzobon, Michele Tessarin, Alberto Pilotto

**Affiliations:** 1 Primary Care Department, Azienda ULSS 3 (Unità Locale Socio Sanitaria) “Serenissima” Veneto Region, Dolo-Mirano District, Venice, Italy; 2 Geriatrics Section, Department of Medicine, University of Palermo, Palermo, Italy; 3 Parc Sanitari Sant Joan de Déu, CIBERSAM, Dr Antoni Pujadas, 42, 08830 Sant Boi de Llobregat, Barcelona Spain; 4 ICREA, Pg. Lluis Companys 23, Barcelona, Spain; 5 Social Unit, Azienda ULSS 3 (Unità Locale Socio Sanitaria) “Serenissima” Veneto Region, Venice, Italy; 6 Social Cooperatives Consortium “G. Zorzetto”, Venice, Italy; 7 Social Direction, Azienda ULSS 3 (Unità Locale Socio Sanitaria) “Serenissima” Veneto Region, Venice, Italy; 8 Sanitary Direction, Azienda ULSS 3 (Unità Locale Socio Sanitaria) “Serenissima” Veneto Region, Venice, Italy; 9 Department Geriatric Care, Orthogeriatrics and Rehabilitation, Frailty Area, E.O. Galliera Hospital, Genoa, Italy; 10 Department of Interdisciplinary Medicine, Aldo Moro University of Bari, Bari, Italy

**Keywords:** COVID-19, Nursing home, Prognosis, Multidimensional prognostic index, Frailty comprehensive geriatric assessment

## Abstract

**Aim:**

Coronavirus-19 disease (COVID-19) is a widespread condition in nursing home (NH). It is not known whether COVID-19 is associated with a higher risk of death than residents without COVID-19. Therefore, the aim of this study was to assess whether COVID-19 is associated with a higher mortality rate in NH residents, considering frailty status assessed with the Multidimensional Prognostic Index (MPI).

**Methods:**

In this retrospective study, made in 31 NHs in Venice, Italy, the presence of COVID-19 was ascertained with a nasopharyngeal swab. Frailty was evaluated using the MPI, modified according to the tools commonly used in our NHs. A Cox’s regression analysis was used reporting the results as hazard ratios (HRs) with 95% confidence intervals (CIs), using COVID-19 as exposure and mortality as outcome and stratified by MPI tertiles. Similar analyses were run using MPI tertiles as exposure.

**Results:**

Overall, 3946 NH residents (median age = 87 years, females: 73.9%) were eligible, with 1136 COVID-19 + . During a median follow-up of 275 days, higher values of MPI, indicating frailer people, were associated with an increased risk of mortality. The incidence of mortality in COVID-19 + was more than doubled than COVID-19- either in MPI-1, MPI-2 and MPI-3 groups. The presence of COVID-19 increased the risk of death (HR = 1.85; 95% CI 1.59–2.15), also in the propensity score model using MPI as confounder (HR = 2.48; 95% CI 2.10–2.93).

**Conclusion:**

In this retrospective study of NH residents, COVID-19 was associated with a higher risk of all-cause mortality than those not affected by COVID-19 also considering the different grades of frailty.

## Introduction

The spread of coronavirus-19 disease (COVID-19) is becoming unstoppable meeting the necessary epidemiological criteria to be declared a pandemic [1]. We know that COVID-19 is caused by the SARS-CoV 2, a type of coronavirus, of which the first human infection was identified in Wuhan province, China [2]. At the end of March 2021, more than 124 million people were officially affected by COVID-19, with more than 2.7 milion deaths in the world [3]. The epidemiological data so far indicated that COVID-19 could be considered as a condition typical of older people [4]. In fact, mortality rates are extremely high in older persons and the prevalence of COVID-19 is higher in older persons compared to the younger ones [5].

A particular interest was given to the COVID-19 outbreak in nursing homes (NHs) for several reasons [6–8]. First, NHs commonly include people that can be considered frail (e.g. for the presence of severe dementia) or disabled [9]. Moreover, these structures have a high proportion of people with relevant social problems, e.g., they are without relatives or they cannot live alone at their homes since they are disabled. All these conditions are, per se, considered to increase mortality risk. Finally, even if less than 10% of all COVID-19 cases are observed in NH in the world, NH and assisted living facilities residents and staff accounted for more than one third of the all deaths recorded [10, 11].

Given this background, it is important to precisely estimate the weight and the importance of COVID-19 in increasing mortality not only in community-dwelling older people, but also in NH setting. For example, in young persons, the COVID-19 pandemic was associated with a significant increase in mortality, being similar to the unintentional opioid overdoses occurring during 2018 in the United States [12]. Similar figures are present for older people [13]. Unfortunately, these epidemiological information are not available for NH people, a setting in which COVID-19 is a widespread condition, as mentioned before [14]. Moreover, the data available so far have shown the importance of frailty for prognosis in older people having COVID-19 [15–17], but these data are mainly based on community-dwelling and hospitalized older people and not in NHs [18–20].

The aim of this study was to assess whether COVID-19 is associated with a higher mortality rate in older persons living in NH, also taking in consideration frailty status assessed with the Multidimensional Prognostic Index (MPI), a common tool for stratifying prognosis and for clinical decision-making in geriatric medicine [21].

## Methods

### Participants

For the aims of this work, we considered all NH residents in the area of Venice, Italy. This entity is in an area of 1406 square Km in Veneto Region, North-East Italy, with about 650,000 inhabitants. This area includes 31 NHs hosting approximately 3850 beds.

At the end of March in response to the increased local awareness of COVID-19 in NH setting, the Veneto Region proposed periodical screening assessments with portable nasopharyngeal swabs for both residents and NH personnel [22]. The period of which study referred was from 01st March to 31st December 2020.

The study was approved by our local Ethical Committee.

### COVID-19 diagnosis

A nasopharingeal swab test with an RT-PCR assays (Copan UTM System, Copan, Italy) for the identification of SARS-CoV-2 was administered to all NH residents.

### Defining frailty using the MPI

A version of the MPI, slightly modified from the original version [23], using tests commonly used in our NHs for clinical follow-up of the residents was used. [24, 25]

Briefly, 9 domains, including 55 different questions, were considered: (1) age, (2) sex, (3) main diagnosis, (4) nursing care needs (VIP), (5) cognitive status (VCOG), evaluated by the Short Portable Mental Status Questionnaire (SPMSQ) [26], (6) pressure sores risk (VPIA), evaluated by the Exton-Smith Scale [27], (7) activities of daily living (VADL) and (8) mobility (VMOB) evaluated by the Barthel Index [28], and (9) social support (VSOC) [29]. To calculate this MPI, we used a weighted sum of each individual domain, taking as outcome mortality after 1 year [29].

We finally divided the participants according to two MPI cut-offs in tertiles, i.e. 0.31 and 0.40 considering in MPI-1 (robust) the lowest tertile (MPI 0–0.31), MPI-2 (pre-frail) the middle tertile (MPI 0.31–0.40) and MPI-3 (frail) the highest tertile (MPI > 0.40).

### Outcomes

The primary outcome of our research was mortality. For NH residents positive to COVID-19, the follow-up period was calculated from the positivity to the nasopharingeal swab test until the date of death or the last observation made on 31st December 2020. For NH residents, the date of the beginning was posed on 01st March. The data regarding mortality are collected routinely as administrative data. The follow-up period was in median = 275 days, ranging from 0 to 295 days.

### Statistical analysis

Continuous variables were evaluated in term of means and standard deviation (SD), after checking their normality. For categorical variables, relative frequencies (%) were reported. Parametric univariate tests (*p* values were referred to Fisher exact for frequencies and *t* Test for means) were used for evaluating possible association according to positivity or not to COVID-19.

The association between COVID-19 and mortality was made using different approaches. First, we reported the incidence of the outcome of interest, per 1000 persons-days overall and in subjects with different grade of frailty, as assessed by MPI. Moreover, we assessed the effect of COVID-19 with mortality using a Cox’s regression analysis, unadjusted and using a propensity score model with age, sex, nursing care needs, cognitive status, pressure sores risk, activities of daily living, mobility, social support, the needing of care assistants, the main medical diagnosis, using a 1:1 nearest-neighbor propensity score matching. The covariate balance for the treated and matched control groups was tested by Student’s t tests and Chi-squared tests for continuous and categorical variables, respectively. Multivariable Cox regression analysis, adjusting for propensity score quintiles, was conducted to assess the association between COVID-19 and mortality. The results were consequently reported as hazard ratios (HRs) with their 95% confidence intervals (95%CI). Similar analyses were run, by MPI tertiles used as stratifying factor and as exposure.

All analyses were performed using the SPSS 21.0 for Windows (SPSS Inc., Chicago, Illinois). All statistical tests were two-tailed and statistical significance was assumed for a *p* value < 0.05.

## Results

### Sample selection

Among 4316 NH residents, 51 aged less than 60 years, 318 did not have sufficient data for MPI calculation, and 1 participant did not report data regarding mortality. Finally, 3946 NH residents (median age = 87 years, range: 60–105; females: 73.9%) were eligible.

### Baseline characteristics

Among 3946 participants initially enclosed, 1136 reported a diagnosis of COVID-19 (overall prevalence rate = 28.8%).

Table 1 shows the baseline characteristics according to the presence or not of COVID-19. NH residents with COVID-19 did not differ in terms of mean age (*p* = 0.85) or female sex (*p* = 0.08) compared to people never affected by COVID-19 (*n* = 2810). Moreover, the prevalence of dementia, immobilization syndrome and cardiovascular disease was similar between COVID-19 positive and negative. NH residents with COVID-19 did not differ compared to their counterparts in any of the MPI domain (activities of daily living, nursing care needs, mobility, pressure sore risk, social support, cognitive status) leading to a similar MPI score (0.36 ± 0.13 in COVID-19 + vs. 0.35 ± 0.13 in COVID-19−, *p* = 0.46) (Table 1).

**Table 1:** Descriptive analysis of nursing home residents, by presence of COVID-19

Domain	COVID-19 − (*n* = 2810)	COVID-19 + (*n* = 1136)	*P* value
Age	86.1 (7.9)	86.1 (7.8)	0.85
Female sex (%)	73.1	75.9	0.08
Dementia (%)	35.2	38.4	0.07
Immobilization syndrome (%)	18.4	20.4	0.10
Cardiovascular disease (%)	11.5	10.1	0.18
VIP	3.24 (5.78)	2.95 (5.15)	0.18
VPIA	4.15 (5.33)	4.42 (5.64)	0.15
VCOG	7.00 (2.86)	7.01 (2.80)	0.87
VADL	49.9 (13.5)	49.8 (13.62)	0.82
VMOB	33.1 (10.1)	33.2 (10.1)	0.97
VSOC	238 (11)	239 (9)	0.08
MPI	0.36 (0.13)	0.35 (0.13)	0.46

*MPI* Multidimensional Prognostic Index, *VADL* activities of daily living, *VCOG* cognitive functions, *VIP* nursing care needs, *VMOB* mobility, *VPIA* pressure sores risk, *VSOC* social support network

### Mortality data

**Fig. 1:**
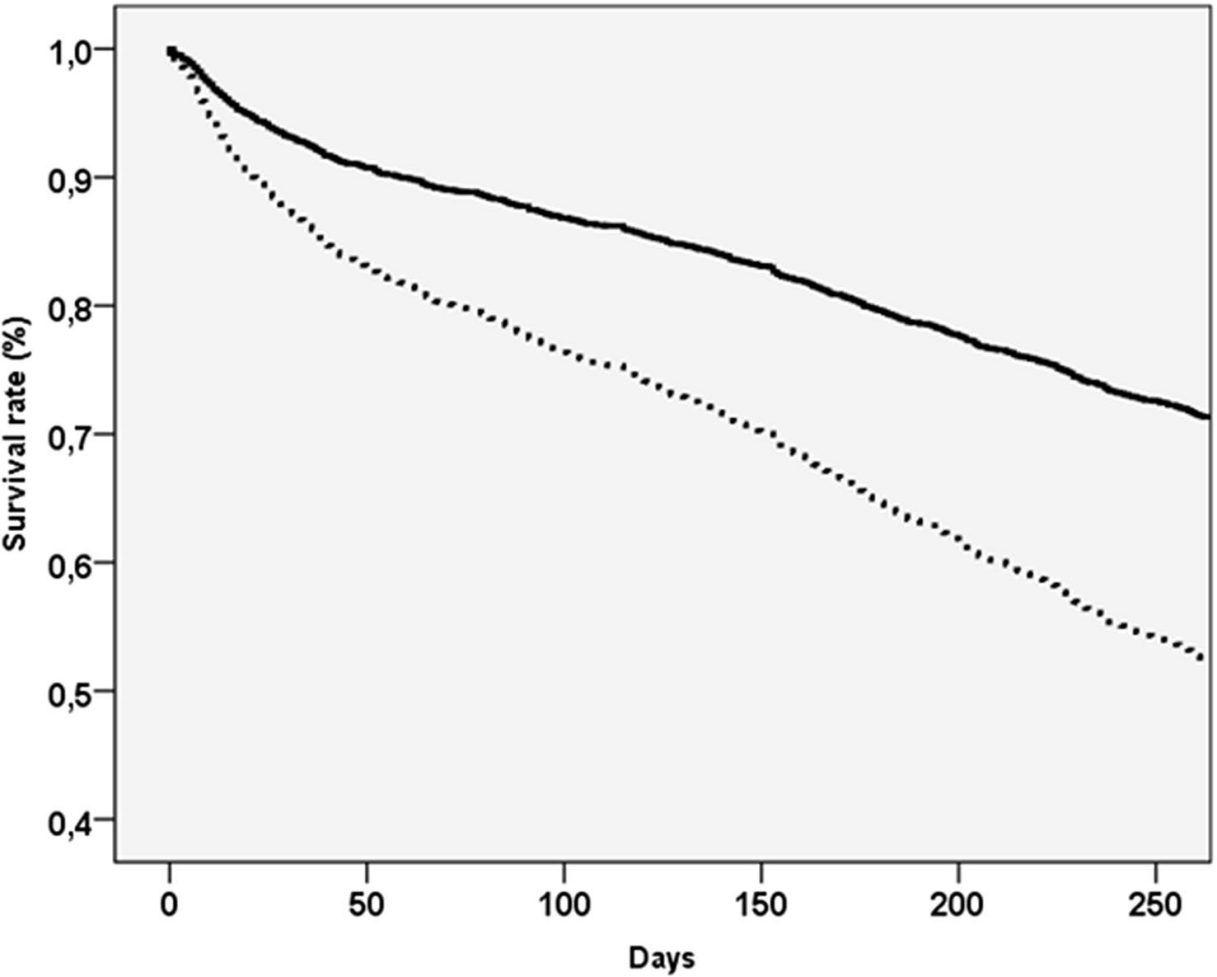
Survival curves, taking mortality as outcome, in the sample as whole for by presence (dashed line) or absence (continuous line) of COVID-19

During the follow-up period that was in median 275 days, we recorded 1187 deaths. Table 2 shows the mortality data according to COVID-19 status. The incidence rate of mortality in people affected by COVID-19 + was more than doubled than in those with COVID-19− (3.00 vs. 1.27 per 1000 persons-days, *p* < 0.0001). In the sample as whole, the presence of COVID-19 increased the risk of death of 85% in the unadjusted model (HR = 1.85; 95%CI 1.59–2.15) and of 148% in the propensity score model (HR = 2.48; 95% CI 2.10–2.93). These findings are graphically reported in Fig. 1.

**Table 2:** Overall and subgroup analyses for nursing home residents by COVID-19 status, taking mortality as outcome: multivariate and propensity score quintiles adjusted models

	COVID-19 −			COVID-19 +			Estimates^b^			
Frailty status^a^	Number of deaths	Number of subjects	Incidence rate (per 1000) (95% CI)	Number of deaths	Number of subjects	Incidence rate (per 1000) (95% CI)	Unadjusted estimate (HR, 95%CI)	*p* value	Propensity Score model^c^ (HR, 95%CI)	*p* value
All sample	901	2810	1.27 (1.19–1.36)	286	1136	3.00 (2.66–3.37)	1.85 (1.59–2.15)	< 0.0001	2.48 (2.10–2.93)	< 0.0001
Robust (MPI lowest tertile)	242	918	1.00 (0.89–1.13)	83	398	2.57 (2.07–3.18)	2.02 (1.50–2.72)	< 0.0001	1.91 (1.37–2.66)	< 0.0001
Pre-frail (MPI middle tertile)	289	944	1.21 (1.08–1.36)	102	371	3.11 (2.55–3.80)	2.07 (1.60–2.68)	< 0.0001	2.90 (1.73–4.86)	< 0.0001
Frail (MPI highest tertile)	370	948	1.60 (1.45–1.78)	101	367	3.33 (2.74–4.07)	1.60 (1.25–2.04)	< 0.0001	2.03 (1.38–2.99)	< 0.0001

^a^Frailty status was assessed using multidimensional prognostic index score tertiles values

^b^Data are reported as hazard ratios (HRs) and their 95% confidence intervals (CIs)

^c^Propensity score was included in quintiles and based on multidimensional prognostic index (MPI) score that includes as domains age, sex, nursing care needs (VIP), cognitive status (VCOG), pressure sores risk (VPIA), activities of daily living (VADL), mobility (VMOB), social support (VSOC), the needing of care assistants (VIP), the main medical diagnosis

Table 2 reports the data by COVID-19 status, according to frailty status. First, the incidence of mortality increased by frailty status, independently from the COVID-19 status (from 1.00 to 1.60 per 1000 persons-days in COVID-19− and from 2.57 to 3.33 per 1000 persons-days in COVID-19 +). Moreover, the presence of COVID-19 leads to an increased risk of death in all the MPI tertiles in both unadjusted (p for interaction across tertiles = 0.38) and propensity score models (*p* for interaction across tertiles = 0.26).

**Fig. 2:**
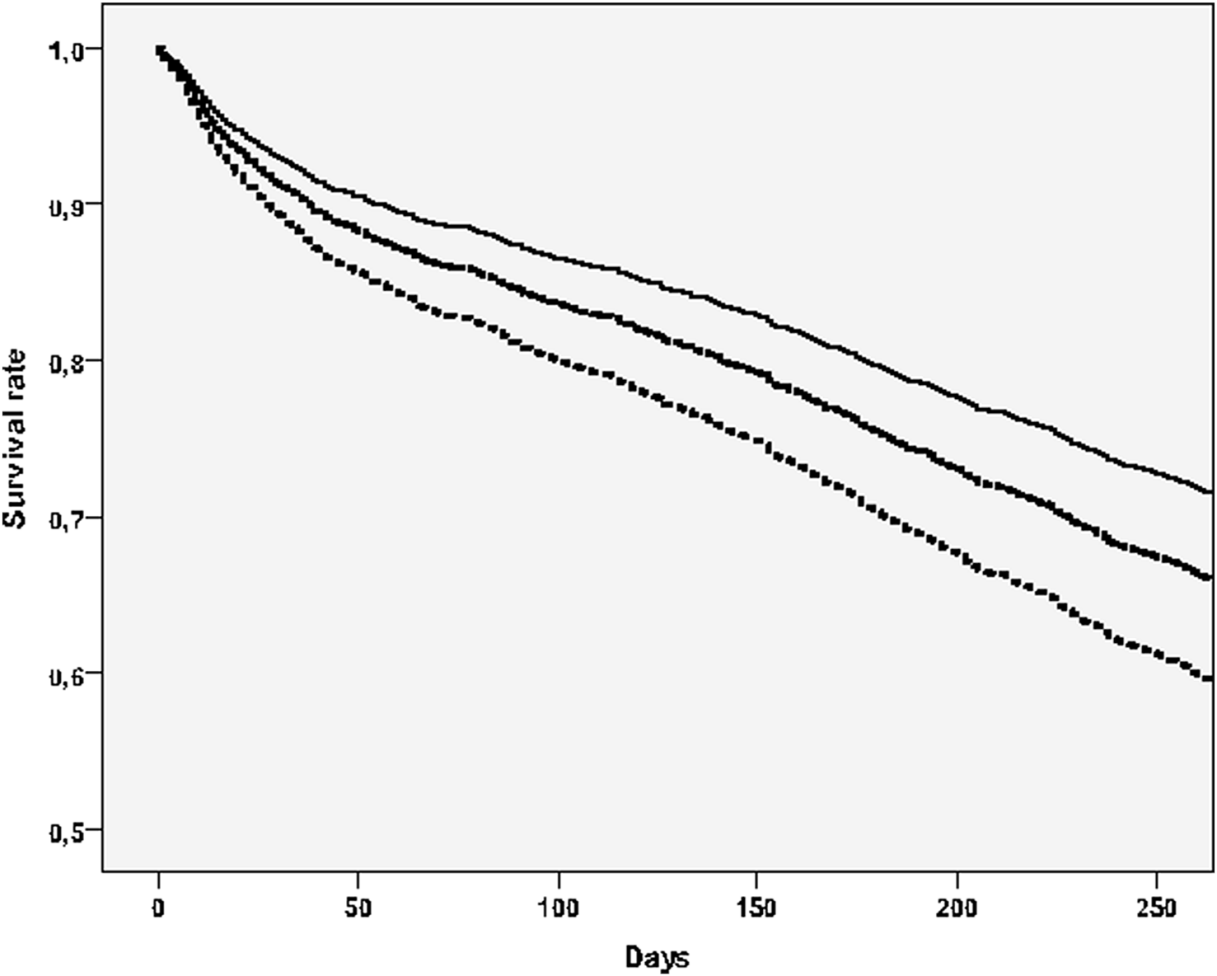
Survival curves, taking mortality as outcome, by multidimensional prognostic index tertiles. Notes: the highest line indicated participants in the lowest MPI group (robust), the lowest those in the highest MPI group (frail)

Finally, our study showed that frailty is associated with an increased risk of death in NH residents. As reported in Fig. 2, taking people in the lowest tertile of MPI as reference and after adjusting for the diagnosis of COVID-19, people in the middle (HR = 1.54; 95% CI 1.34–1.78; *p* < 0.0001) and in the highest (HR = 1.89; 95% CI 1.65–2.17; *p* < 0.0001) tertile carried a significant higher risk of mortality.

## Discussion

In this research, we found that in NH setting COVID-19 was associated with a significant higher risk of mortality, also when considering the presence of frailty, as estimated with the MPI score. Moreover, our paper further confirmed that frailty, as assessed by the MPI, increased the risk of mortality in a special context, such as NH.

Even if it is known that COVID-19 mortality linearly increases with age, limited evidence is available regarding the fact is COVID-19 is able or not to increase mortality in older persons resident in NH that can be considered frail per se. In this regard, few studies using resident-level data are available, often including data from a single facility or a small number of facilities, therefore, limiting the generalizability of these findings [14, 30–32]. Even if all these researches advanced our knowledge regarding mortality in NH during COVID-19 some limitations should be discussed. The most important is that these works did not have any control group; therefore, it is not possible to know if COVID-19 is able or not to increase mortality in this population characterized by a high mortality rate. Moreover, these papers did not consider the presence of a comprehensive geriatric assessment (CGA) tool, such as the MPI as we did. In our study, we tried to overcome these shortcomings including a control group never affected by COVID-19 during the follow-up period and comparable for the level of frailty. Our study, for the first time, showed that COVID-19 prevalence is not dependent on frailty and that COVID-19 leads to an increased risk of mortality not only in frailer subjects, but also in more robust ones. Our analyses, in fact, showed that people with and without COVID-19 are similar not only in terms of age, gender and comorbidities, but also relevant characteristics such as nursing care needs, cognitive status, pressure sores risk, activities of daily living, mobility, social support, and the needing of care assistance. Overall, our findings suggest that the presence of COVID-19 in NH practically doubled the risk of death in this setting, also when taking in account frailty, as evaluated by MPI.

To know that COVID-19 is able to significantly increase the risk of death in NH setting is, in our opinion, of clinical importance. Before our study, in fact, the sensation of several authors was that COVID-19 leaded to mortality only because frailer people were affected by this condition [30]. On the contrary, our study shows that COVID-19 is present in NH residents independently from the presence of frailty since the MPI score, at the baseline evaluation, was similar between COVID-19 + and COVID-19− residents. Moreover, the adjustment for a multidimensional score attenuated, but not nullified the association between COVID-19 and mortality in NH setting, indicating that COVID-19 is, unfortunately, an important cause of death in this population, independently from frailty.

From an epidemiological perspective, individuals resident in NH (such as in our case) are affected by advanced dementia (about one over three in our sample) and immobilization syndrome that often are present with dysphagia, associated with an increased risks of malnutrition, aspiration, bacterial pneumonia, and delirium, that further complicate the course of COVID-19 [14]. As widely known, these subjects generally require extensive assistance with the activities of daily living that put them close with many staff members who may be asymptomatically infected with viral strains from the community. [14, 33] However, again, our study suggests that the prevalence of COVID-19 is not dependent from the necessity of assistance in the NH (e.g., the Barthel Index score was similar between COVID-19 + and negative), suggesting that other research should be done regarding the risk factors for the difference in prevalence in NH.

Our findings should be considered in the light of certain limitations. First, the cause of death was not included in our analyses. Therefore, it is not possible to know the reasons why people affected by COVID-19 died more than their counterparts. Second, our study population resident in NH, who are traditionally frailer than people living in the community; therefore, our findings are not applicable to community-dwelling adults. Third, we did not have any information regarding the severity and the eventual therapy used in NHs, being administrative data. Fourth, even if we collected information until 31st December 2020, our NHs and Italy in general are assisting to a third wave of COVID-19 that can modify our findings. Fifth, our study is a retrospective research: therefore, a selection bias cannot be excluded.

In conclusion, in this retrospective study of Italian NH residents, COVID-19 was associated with a higher risk of all-cause mortality than those not affected by this condition, also in case of similar frailty scores. Our findings further suggest the importance to prevent the presence and the diffusion of COVID-19 in NHs that is associated per se with a high rate of mortality in this setting. Other prospective studies are needed to confirm our findings.

## Data Availability

Available upon reasonable request to the corresponding author.

## Code availability

Available upon reasonable request to the corresponding author.
